# Clinical and molecular cytogenetic analyses of four patients with imbalanced translocations

**DOI:** 10.1186/s13039-016-0244-x

**Published:** 2016-04-19

**Authors:** Hong Yan Liu, Jia Huang, Tao Li, Dong Wu, Hong Dan Wang, Yue Wang, Tao Wang, Liang Jie Guo, Qian Nan Guo, Fei Fei Huang, Rui Li Wang, Ying Tai Wang

**Affiliations:** Department of Medical Genetics Institute, People’s Hospital of Zhengzhou University (Henan Provincial People’s Hospital), Zhengzhou, 450003 Henan China; Department of Gynaecology and Obstetrics, People’s Hospital of Zhengzhou University (Henan Provincial People’s Hospital), Zhengzhou, 450003 Henan China; Department of Ultrasonography, People’s Hospital of Zhengzhou University (Henan Provincial People’s Hospital), Zhengzhou, 450003 Henan China

**Keywords:** Imbalanced translocation, Intellectual disability, Delayed growth, Language barrier, Chinese

## Abstract

**Background:**

Chromosomal abnormalities that result in genomic imbalances are main causes of congenital and developmental anomalies including intellectual disability and multiple congenital malformations. In this report we describe four patients from three families with imbalanced translocations. Only a small percentage of imbalanced translocation individuals can be born to live, most of them were aborted in embryonic period. It is of great significances to precisely analysis the chromosome variation to study the relationship between genotype and phenotype.

**Results:**

Four patients showed common clinical manifestations including delayed growth, intellectual disability, language barrier and facial dysmorphisms. In addition to the above features, lower limbs dysplasia and both foot eversion were found in patient 1, brachydactylic hand, cerebellar ataxia and congenital heart defects were also found in patient 4. Conventional karyotype analysis revealed abnormal karyotypes 46, XX, der (6) t (6: 10) (p23; q24), 46, XX, der (20) t (3; 20) (p23; p13) and 46, XX, der (22) t (3; 22) (q27; q13.3) in the four patients, respectively. Array-CGH analyses confirmed 23.6 Mb duplication on 10q25.1-q26.3 and 0.9 Mb deletions on 6p25.3, 19.9 Mb duplication on 3p24.3-p26.3 and 0.25 Mb deletion on 20p13 and 12.5 Mb duplication on 3q27.2-q29 and 1.9 Mb deletions on 22q13.2-q13.33 in the four patients, respectively.

**Conclusion:**

Parents with balanced translocation are passed the imbalanced chromosome to patient, and the partial monosomy and partial trisomy lead to multiple congenital malformations of four patients.

**Electronic supplementary material:**

The online version of this article (doi:10.1186/s13039-016-0244-x) contains supplementary material, which is available to authorized users.

## Background

Chromosomal abnormalities that result in genomic imbalances are main cause of congenital and developmental anomalies including intellectual disability and multiple congenital malformations. The abnormal chromosomes of parents can be passed to next generation, particularly for parents carrying a balanced translocation or inversion. Balanced chromosomal translocations are often identified as a cause of infertility [1], owing to meiotic impairment can lead to gamete formation problems, in man can cause failure of spermatogenesis [1, 2]. Research found that most breakpoint of balanced chromosome translocation was at or near the localization of segmental duplications which contribute to genomic instability [3], so the genetic disorders may increase of the offspring. Theoretically, for balanced translocation carriers, the chance of passing the imbalanced chromosome to child is 16/18, whereas passing a normal chromosome and balanced chromosome is 1/18, respectively. The former case will lead to either a spontaneous miscarriage or a live born child with intellectual disability and/or multiple congenital malformations. The reported live births rate of couple with balanced translocations ranges from 14 to 19.5 % [4, 5], and more than 80 % of fertilized eggs were spontaneous abortion because of aneuploidy, polyploidy, imbalanced translocation and other reasons [5]. In clinic, half of these babies with chromosome abnormalities were inherited from a parent carrying balanced translocation or inversion [6].

Precise characterization of the chromosomal rearrangements and identification of the associated genes are important for syndrome recognition and genetic risk assessment. Array-CGH is a powerful tool for the accurate detection of genomic imbalances. It enables identification of the genes that cause mental retardation when abnormal copy numbers exist [7]. In this report we present three families with imbalanced translocation. All families had offspring with mental retardation who carried imbalanced translocation. We provided detailed analysis of the genotype-phenotype correlation and discussed possible candidate genes.

## Methods

### Participants

**Fig. 1:**
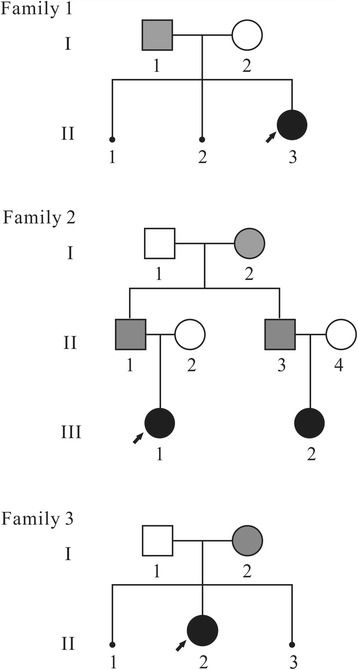
Pedigree of three families reported in this study. *Black symbols* indicate imbalanced translocation individuals; *grey symbols indicate* balanced translocation individuals; *black spot* indicate abortion

Four Patients were from three Chinese families without family history of congenital malformations. Patients showed common clinical manifestations, such as delayed growth, intellectual disability, language barrier and facial dysmorphisms. Parents of these patients went to Medical Genetics Institute for genetic consulting. Routine clinical examination was performed on four patients. Detailed birth, medical and clinical manifestations were collected after informed consent was obtained. The pedigree of three families was shown in Fig. 1.

All examinations were performed according to the guidelines of the “declaration of Helsinki”. Ethical approval was obtained from the People’s Hospital of Henan Provincial Ethics Committee.

### Karyotyping and array-CGH analyses

Five milliliter peripheral venous blood was collected from four patients and their parents; 20 ml amniotic fluid was collected from mother of patient 4.

Karyotyping on GTG-banded chromosomes from patients and their parents was performed on cultured lymphocytes (patient 1 to 4 and their parents) and cultured amniocytes (family 3 II2) according to standard protocols.

To precisely determine the cytogenetic variation, a high-resolution chromosomal analysis was performed using a whole-genome oligonucleotide CGH microarray. Genomic DNA from four patients was extracted from peripheral blood lymphocytes using Qiagen blood kit (QIAGEN, Germantown, MD, USA).

Array-CGH was performed using an Agilent SurePrint G3 Human CGH Microarray Kit, 8x60K G4450A whole-genome oligonucleotide microarray (41 kb average probe spacing; 33 kb in Refseq genes). Digestion, ligation, PCR, Cy5-dUTP and Cy3-dUTP labeling, hybridization of test and reference DNA were performed according to the manufacturer’s recommendations (Agilent Technologies, Santa Clara, California, USA). Slides were scanned using an Agilent SureScan Microarray Scanner, and analyzed using Agilent CytoGenomics 2.9. Significant copy-number changes were identified by at least three consecutive aberrant probes. Reference human genome was GRCh36/hg18.

## Results

### Family 1

The patient 1 (family 1: II3) is a 3 years old female. She was from the third pregnancy of healthy and nonconsanguineous young parents. Her mother had a history of two miscarriages. The physical examination revealed that patient 1 presented with multiple congenital anomalies. She had significant facial dysmorphic features, including depressed nasal bridge, anteverted nostrils, long philtrum, downturned angles of mouth and thin upper lips, carp-shaped mouth, high arched palate, micrognathia, low set ears and low anterior hairline. Developmental evaluations showed delayed growth with short stature (85 cm, <3rd centile) and low weight (9.5 kg, <3rd centile). She walked without assistance at 2 years and 3 month of age, presented with walking instability. Limbs dysplasia included slim lower limbs and foot eversion on both sides. Neurological exam revealed muscular hypotonia, nerve reflex insensitivity, intellectual disability and language barrier. Feeding difficulty was present in early infancy. The patient has no history of seizure activity and congenital heart defect. Hearing, vision, and hands were normal.

**Fig. 2:**
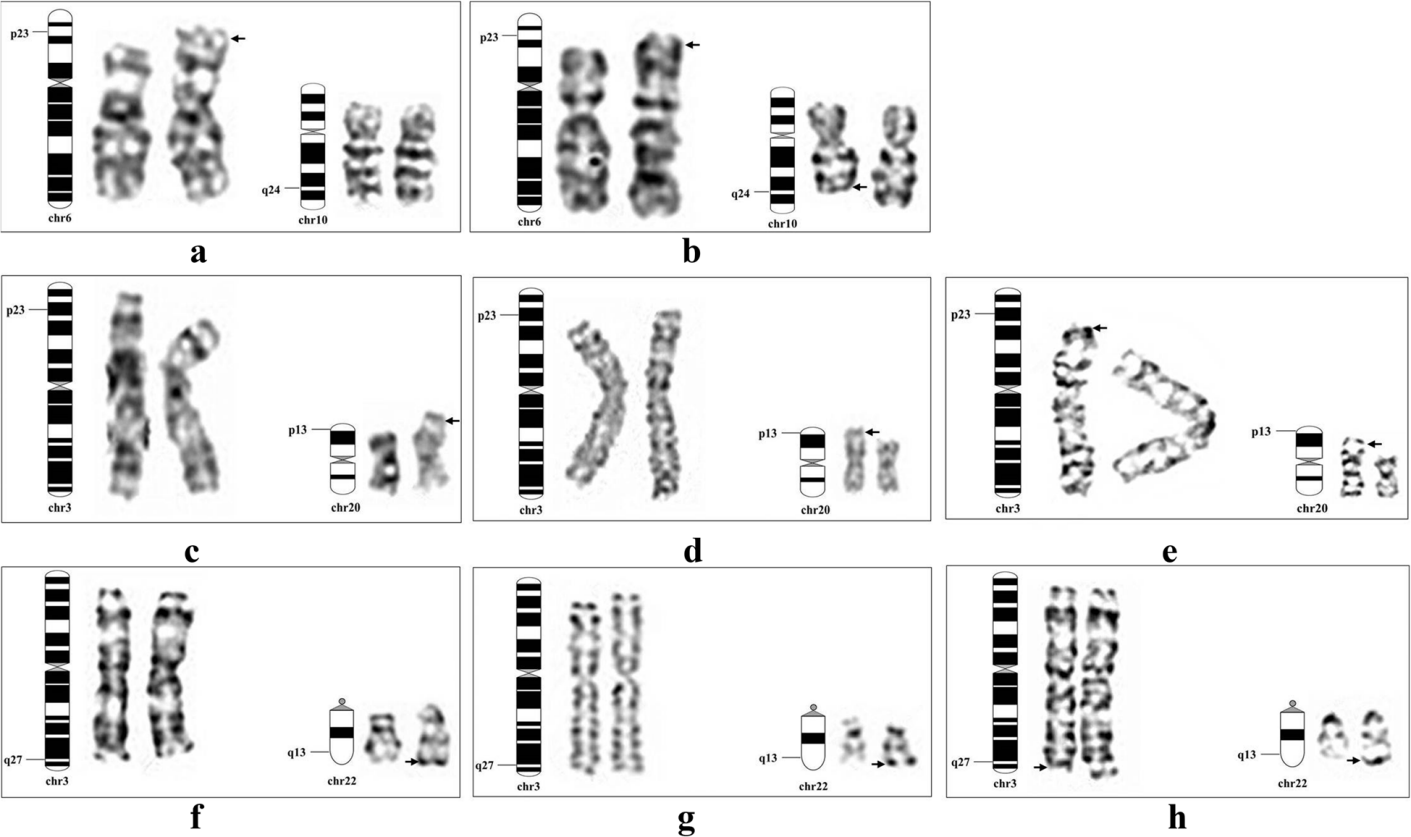
GTG-banding karyotype analysis image of patients and their parents. Family 1 was shown in upper panel. **a** and **b** were patient 1 and father, respectively; Family 2 was shown in middle panel. **c**, **d** and **e** were patient 2, 3 and father, respectively; Family 3 was shown in lower panel. **f**, **g** and **h** were patient 4, fetus and mother, respectively

The patient karyotyping was performed on peripheral blood lymphocytes. The report revealed abnormal karyotype 46, XX, der (6) t (6: 10) (p23; q24) (Fig. 2a). A similar protocol was followed for her parents’ chromosome preparation. The report revealed normal mother with 46, XX pattern, but the father had balanced translocation with 46, XY, t (6; 10) (p23; q24) pattern. This chromosomal abnormality was a balanced translocation between 6 and 10 (Fig. 2b).

**Fig. 3:**
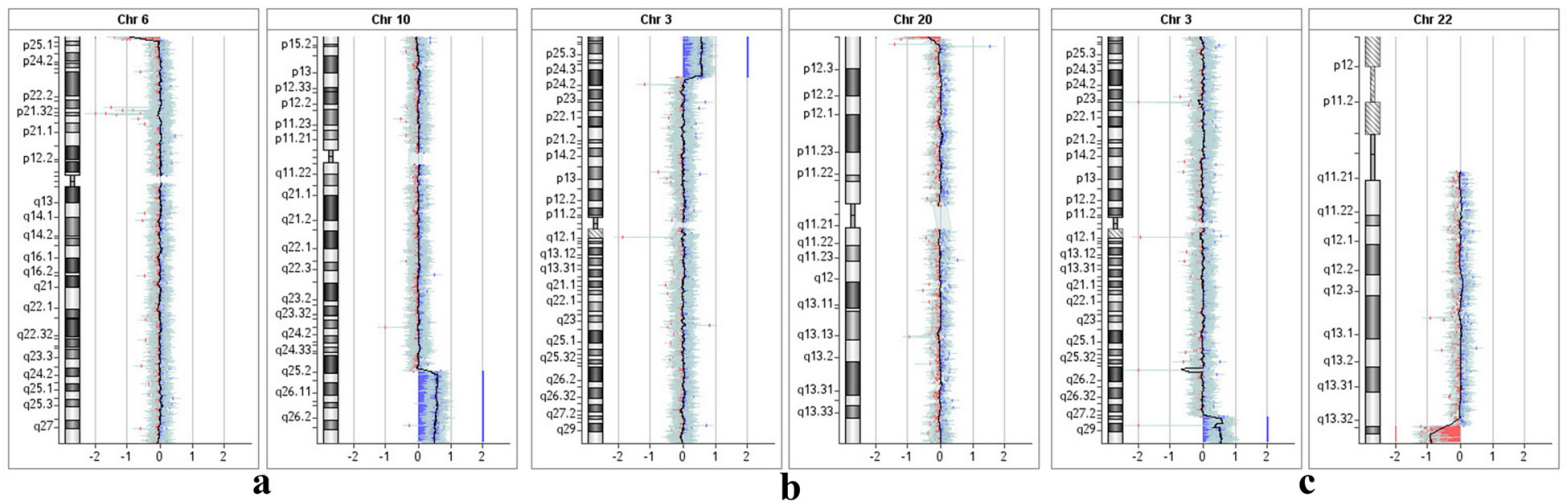
Array-CGH analysis showed telomeric rearrangements on two different chromosomes in four patients (**a**: patient 1; **b**: patient 2 and 3; **c**: patient 4). The log ratio was reported (log2 intensity of [Cy5 fluorochrome/Cy3 fluorochrome)] on the X-axis. Expected values were from −0.7 to −1 for a deletion (*red color*), 0 for normal (*black line*), and +0.5 to +1 for a duplication (*blue color*)

Array-CGH analysis was performed on lymphocytes DNA of this patient and detected a 23.6 Mb duplication at 10q25.1-q26.3 (chr10:111770926–135404523) and a 0.9 Mb deletion at 6p25.3 (chr6:170426–1106808) (Fig. 3a).

### Family 2

In this family, two female cousins (patient 2 and 3) were found with delayed growth, intellectual disability and language barrier. Although with normal weight, their heights were lower than peers. The older cousin (patient 2), a 9 years old girl, also showed facial dysmorphic features, including depressed nasal bridge, ocular hypertelorism, broad nose, thin upper lip, round face with full cheeks and facial expression impairment. She could express some simple language, and have learning difficulties. She had normal motor system with good coordination and could play freely. The patient 2 had no history of seizures and cardiomyopathy. She had normal hearing and vision, as well as normal finger nails. The younger cousin (patient 3), a 4 years old girl, has facial dysmorphic features, including open mouth, thin upper lip and facial expression impairment. She could not speak any meaningful words. Other clinical manifestations were same as her older cousin.

Conventional karyotype analysis showed that two female cousins carried 46, XX, der (20) t (3; 20) (p23; p13) (Fig. 2c and d). The old cousin parents accepted the subsequent karyotyping with the report revealing normal 46, XX pattern of her mother, but the father had balanced translocation 46, XY, t (3; 20) (p23; p13) (Fig. 2e). The younger cousin parents and grandmother were also karyotyped. The report showed balanced translocation of 46, XX/XY, t (3; 20) (p23; p13) in the father and grandmother. Their GTG-banding images were not available.

Array-CGH analysis was performed in two female cousins to further characterize the rearrangement. This analysis identified that two female cousins have same abnormality rearrangement with a 19.9 Mb duplication at 3p24.3-p26.3 (chr3:93949–19992330) and 0.25 Mb deletion at 20p13 (chr20: 121521–373182) (Fig. 3b).

### Family 3

In this family, the healthy and nonconsanguineous young couples experienced three pregnancies history. After one miscarriage, they had a baby girl (family3: II2) at term by cesarean section. The couples went to our institution for genetic consulting, when pregnancy again, because their 5 years old daughter showed delayed growth, intellectual disability and language barrier. The girl also had facial expression dementia, paratrichosis (long eyelashes, bushy eyebrows and hair), brachydactylic hand, hypoplasia of distal phalanges, single palmar crease bilaterally, and thumb flat abduction of 90°. She walked independent at 3 years of age, but presented with cerebellar ataxia and walking instability. The girl could not finish fine motor coordination. She was not able to express simple language, speak slowly and ambiguously, and could only call mom and dad. Neurological exam revealed muscular hypotonia, nerve reflex insensitivity. She suffered congenital patent ductus arteriosus which was surgically cured at 2 years old. The hearing and vision was normal. Prenatal ultrasonography examination for the new pregnancy showed that fetus had bilateral multiple choroid plexus cyst with a larger size (3.3 * 3.3 mm) on the right side. The diameter of right renal pelvis was 4.5 mm. Thickening of the placenta and single umbilical artery was also present.

Karyotype analysis showed that the girl and fetus had same karyotyped 46, XX, der (22) t (3; 22) (q27; q13.3) (Fig. 2f and g). Their parents were also karyotyped with the report showing normal 46, XY in the father, but abnormal 46, XX, t (3; 22) (q27; q13.3) in the mother (Fig. 2h).

Array-CGH analysis on the peripheral blood DNA from the girl showed a 12.5 Mb duplication at chromosome 3q27.2-q29 (chr3:185307872–197840339), and a 1.9 Mb deletion at 22q13.2-q13.33 (chr22: 49286759–51178264) (Fig. 3c).

## Discussion

In this work, we report three families with balanced translocation. They all have imbalanced translocation offspring with delayed growth, intellectual disability and language barrier. The complex phenotypic manifestation of patients was contributed jointly by monosomy and trisomy of the two chromosomes. Generally, phenotypes associated with deletions are more severe than those associated with duplications. However, because the maximum tolerance in living organisms was different, it is very difficult to justify the predominant factors. By reviewing the published literatures, the prominent phenotypic features of these patients were not classified as some specific syndrome.

The same karyotype has been reported in one patient of India [8]. Conventional karyotype shows patient with 46, XY, der (6) t (6; 10) (p23; q24) pattern which is the same as patient 1. However, genitalia malformation, malformed ears, atrial septal defect and so forth features of India patient were not found in the patient 1. The lower limbs dysplasia was prominent in patient 1, but not reported in India patient. The facial features were similar in the two patients. The different breakpoint may be one of the main reasons that lead to the distinguished characteristics. One patient with 10q25.1-10q26.3 duplication reported by Carter et al. [9] has craniofacial dysmorphia, such as depressed nasal bridge, high arched palate, low set ears and low anterior hairline were same as patient 1, however, skeletal anomalies were present in this patient, including hip dysplasia and mild scoliosis, which were not found in patient 1. Hand anomaly and wide space between the first and second toes (sandal gap) were found in India patient and Carter’s patient, but was not found in patient 1, foot eversion was only present in patient 1. Hearing loss was found in India patient and Carter’s patient, while patient 1 with normal hearing. They all had delayed growth, Intellectual disability and language barrier. We summarized Table 1 for comparison of the clinical features of this patient with similar segmental duplication and/or deletion chromosome. The *FGFR2* was localized in this segmental duplication and was reported to be associated with many syndromes [10]. *FGFR2* is a member of the fibroblast growth factor receptor family and encodes a receptor tyrosine kinase important for osteogenesis. Patients with *FGFR2* missense mutation were mainly manifested as craniofacial and limb abnormalities. Duplication of *FGFR2* may be responsible for the craniofacial features and skeletal anomalies in the above patient. We summarized OMIM gene associated with phenotype localized in this duplicated segment 10q25.1-q26.3 (chr10:111770926–135404523) (Additional file 1). SMC3 mutation have been reported in subjects with Cornelia de Lange syndrome 3 [11], and these patients presented with craniofacial dysmorphia and mental retardation. *SFXN4* mutation was relevant to growth retardation, hypotonia, and speech delay [12]. *BBIP1* and *SHOC2* mutation can lead to mental retardation [13, 14]. Delayed growth, language barrier and neurocognitive developmental impairment may be a result of duplication of these genes.

**Table 1:** The clinical features of patient with similar segmental duplication and/or deletion chromosome

	Patient 1	[8]	[9]	[15]	Patient 2	Patient 3	[16]	[17]	[22]	[23] patient 2	Patient 4	[25]	[27]	[30]	[31]
Segmental duplication/deletion	dup 10q25.1-10q26.3; del 6p25.3	dup 10q24-10qter; del 6p23-6pter	dup 10q25.1-10q26.3^a^	del 6p25.3 -p25.2^b^	dup 3p26.3-p24.3; del 20p13	dup 3p26.3-24.3; del 20p13	dup 3p26.2-p24.1^c^	dup 3p26.2-p25.3	del 20p13^d^	20p13-pter	dup 3q27.2-3q29; del 22q13.32-22q13.33	dup 3q26.3-qter	dup 3q27-qter^e^, del4p16.3^f^	del 22q13.31–q13.33^g^	del 22q13.3^h^
Sex	F	M	F	F	F	F	M	M	F	F	F	M	F	M	F
Age at last follow up (years)	3y	1 m	7y	40y	9y	4y	5y	9y	9y	15y	5y	11 m	4y	5y	14y
Development delay	+	+	+	-	+	+	+	+	+	+	+	+	+	+	+
Craniofacial features															
Ocular hypertelorism	-	+	-	-	+	-	+	-	-	-	-	-	+	-	-
Epicanthic folds	-	+	+	-	-	-	-	-	-	-	-	-	+	+	-
Depressed nasal bridge	+	+	+	-	+	-	+	-	-	-	-	Wide	Wide	-	-
Anteverted nostrils	+	-	-	-	-	-	-	-	-	-	-	+	-	-	-
Long philtrum	+	Broad	Flat	-	-	-	-	-	Flat	-	-	Prominent	+	-	-
Carp-shaped mouth	+	+	-	-	-	-	-	-	-	-	-	-	-	-	-
Thin upper lips	+	-	+	-	+	+	-	-	+	-	-	+	-	-	-
High arched palate	+	+	+	-	-	-	-	-	+	+	-	-	-	-	-
Micrognathia	+	+	-	-	-	-	-	-	-	-	-	+	-	-	-
Low set ears	+	+	+	-	-	-	-	-	-	+	-	+	-	-	-
Malformed ears	-	+	-	-	-	-	prominent ears	-	-	+	-	-	-	-	-
Low anterior hairline	+	-	+	-	-	-	-	-	+	-	-	+	high frontal hairline	-	-
Long eyelashes	-	-	-	-	-	-	-	-	-	-	+	+	-	-	-
Bushy eyebrows	-	-	-	-	-	-	-	-	-	-	+	+	-	-	-
Facial expression anomaly	-	-	-	-	+	+	-	-	-	-	+	-	-	-	-
Microcephaly	-	-	-	-	-	-	+	-	-	-	-	-	-	-	+
Cognitive development															
Intellectual disability	+	+	+	-	+	+	+(mild)	+	+	+	+	+	+	+	+
Language impairment	+	+	+	-	+	+	+	+	-	-	+	N.A	+	+	+
Learning impairment	+	N.A	+	-	+	+	+	+	+	+	+	N.A	N.A	N.A	N.A
Heart defect	-	+	-	-	-	-	-	-	-	-	+	+	-	-	-
Hearing loss	-	+	+	+	-	-	-	-	-	-	-	-	-	-	-
Hand and foot															
Hand anomaly	-	+	+	+	-	-	+	+	+	+	+	+	+	+	-
Foot anomaly	+	+	+	-	-	-	+	-	+	-	-	+	-	-	-
Single palmar crease	-	+	-	-	-	-	+	-	-	-	+	-	-	-	-
Skeletal anomalies															
Limbs	+	-	-	-	-	-	-	-	-	-	-	-	-	-	-
Scoliosis	-	-	+	-	-	-	-	-	-	-	-	-	-	-	-
Hip dysplasia	-	-	+	-	-	-	-	-	-	-	-	+	-	-	-
Other															
Muscular hypotonia	+	-	+	-	-	-	+	-	-	-	+	-	+	+	+
Cerebellar ataxia	-	-	-	-	-	-	-	-	-	-	+	-	-	-	-
Genital malformation	-	+	-	-	-	-	+	+	-	-	-	-	-	-	-
Short neck	+	+	+	-	-	-	+	-	+	-	-	+	-	-	-
Epilepsy	-	-	-	-	-	-	+	-	-	+	-	-	-	+	+
Round face with full cheeks	-	-	-	-	+	-	+	+	-	-	-	-	-	-	-
Central obesity	-	-	-	-	-	-	-	+	-	-	-	-	-	-	-

*F* female, *M* male, *y* years, *m* month, *+* presence of trait, *−* absence of trait, *N.A* not available, a: chr 10: 108 299 681–133 745 613, b: chr6: 203,722 - 2,740,501, c: chr3:3943808–29668053, d: chr20:1–1150000, e: chr3:183097052–198295559, f: chr4:1–769191, g: chr22:46,204,739–51,178,264, h: chr22:49,389,829–51,178,264

One 40-year-old female with 6p25.3 -p25.2 (chr6: 203,722 - 2,740,501) deletion [15], extensive white matter changes is the main clinical feature, normal cognition and intelligence. Patient 1 with 6p25.3 (chr6:170426–1106808) deletion contain 3 Refseq gene, only one OMIM gene (*IRF4*) was associated with Skin/hair/eye pigmentation. Patient 1 was only 3 years old have not presentation these symptoms. Therefore, duplication of 10q25.1-q26.3 was leading role in contribute to the clinical findings seen in patient 1.

Partial trisomy 3p syndrome is characterized by multiple congenital abnormalities, delayed speech, intellectual deficiency and so forth [16]. The clinical manifestations are quite varied, depending on the amount of 3p genomic content in the trisomic state.

Patient 2 and 3 with 19.9 Mb (chr3:93949–19992330) duplication of the 3p24.3-p26.3, main clinical features are development delay, intellectual disability, language impairment and learning impairment. One 5 years old boy with pure 3p26.2-p24.1 duplication reported by Natera-de Benito et al. [17] and one 9 years old boy with pure 3p26.2-p25.3 duplication reported by Bittel et al. [18] share the above clinical malformations. Two genes located in the common segmental duplication of four patients, SUMF1 mutations result in defective post-translational modification of sulfatases, and show association with mental retardation, neurological deterioration, hydrocephalus and so on [19]. Loss of function mutation in SETD5 was associated with mental retardation, intellectual disability, delayed speech and so forth [20]. Duplication of tow genes may be account for development delay and cognitive impairment. Interestingly, the clinical feature of round face with full cheeks was found in patient 2 and two boy patients reported by Natera-de Benito et al. and Bittel et al., and the 9 years old boy presented with central obesity. *GHRL* located in the common segmental duplication of four patients, was known to be associated with obesity [21]. For patient 2 and 3, we will be extraordinarily watchful the possible onset of obesity. It is worth noting that the clinical feature of hand/foot anomaly and genital malformation were not found in patient 2 and 3, however, two boy patients reported by Natera-de Benito et al. and Bittel et al. were presented with above clinical features.

There are reports of individuals with deletion at 20p13 [22–24], we found that the similarly clinical features of these patients are developmental delay and intellectual disability. Patient 2 and 3 with 0.25 Mb (chr20: 121521–373182) deletion of the 20p13, at this segmental deletion contain 10 Refseq genes, not found OMIM gene associated with phenotype, of which two candidate gene (*SOX12* and *NRSN2*) was identified as nervous system expressing genes that may be associated with developmental delay [24].

The clinical manifestations of the 3q syndrome include typical facial features, growth and mental retardation, and frequent congenital heart defects [25]. Patient 4 presented with congenital patent ductus arteriosus, whereas other patient showed ventricular septal defect [25]. Patient 4 had long eyelashes and bushy eyebrows, which was also found in other family with 3q duplication [25, 26]. Patient 4 showed prominent cerebellar ataxia symptom, but the symptom was absence in other family with 3q duplication [25–27]. The feature of hand/foot anomaly was found in Patient 4 and other patients [25–27]. *TP63* located in the common segmental duplication of above patients, and was known to be associated with many syndromes. The clinical features of patient with *TP63* mutation were major presented with hand/foot anomaly and ectodermal dysplasia. *TP63* duplication may be contributed to hand/foot anomaly of these patients. Development delay and cognitive impairment were also found in Patient 4 and other patients [25–27]. Interestingly, the clinical feature of cerebellar ataxia was only found in patient 4. Mutation in *KIAA0226* causes spinocerebellar ataxia [28] with patients presenting progressive incoordination of gait and poor coordination of hands, speech and eye movements, and cerebellar ataxia in early childhood and delayed motor development, as well as dysarthria and hyporeflexia. *KIAA0226* may be a candidate gene for ataxia of patient 4.

The 22q13.3 deletion syndrome, also known as Phelan-McDermid syndrome, this syndrome is characterized by neurological deficits which include global developmental delay, moderate to severe intellectual impairment, absent or severely delayed speech, and neonatal hypotonia. In addition, more than 50 % of patients show autism or autistic-like behavior, and therefore it can be classified as a syndromic form of autism spectrum disorders (ASD) [29], some patients also have seizure manifestations [30, 31], but patient 4 absence of autism and seizure manifestations, cerebellar ataxia is the prominent characteristic different from other patient with the deletion of 22q13.3 [30, 31]. Development delay and cognitive impairment were common features of patient 4 and other patients [30, 31]. *SHANK3* located in the segmental deletion, the *SHANK3* mRNA is localized to proximal and distal dendrites, and is highly expressed the hippocampus, cerebellar granular cells, caudate putamen and thalamic nuclei. It is a key part of normal neuron morphology and connectivity, and play an important role in synaptic formation and function, promote synaptic plasticity, and is critical for learning and memory [32]. Haploinsufficiency of *SHANK3* may be contributed to the development delay and cognitive impairment. *ARSA* encodes the lysosomal enzyme arylsulfatase A, mutation in this gene was reported in metachromatic leukodystrophy, characterized by progressive demyelination, gait disturbances, ataxias, optical atrophy, dementia, seizures, and spastic tetraparesis [33]. *ARSA* may be a candidate gene for ataxia of patient 4.

## Conclusion

The monosomy and trisomy of the two chromosomes lead to complex phenotypic manifestation of patients. These genes (Additional file 1) located in the region of rearrangement chromosome might promote abnormal clinical features of patients. We speculate that other genes may also be involved and multiple factors may play important role in the development of the abnormal features observed in our patients.

### Consent

Written informed consent was obtained from patient’s parents.

## Additional file

Additional file 1: The OMIM gene associated with phenotype localized in the duplicated and deletion segment of four patients. (XLSX 20 kb)
